# Bacterial vaginosis among women at high risk for HIV in Uganda: high rate of recurrent diagnosis despite treatment

**DOI:** 10.1136/sextrans-2015-052160

**Published:** 2015-08-07

**Authors:** Suzanna C Francis, Clare Looker, Judith Vandepitte, Justine Bukenya, Yunia Mayanja, Susan Nakubulwa, Peter Hughes, Richard J Hayes, Helen A Weiss, Heiner Grosskurth

**Affiliations:** 1 MRC Tropical Epidemiology Group, London School of Hygiene and Tropical Medicine, London, UK; 2 MRC/UVRI Uganda Research Unit on AIDS, Entebbe, Uganda

**Keywords:** AFRICA, BACTERIAL VAGINOSIS, COMMERCIAL SEX, CONTRACEPTION, WOMEN

## Abstract

**Objectives:**

Bacterial vaginosis (BV) is associated with increased risk for sexually transmitted infections (STIs) and HIV acquisition. This study describes the epidemiology of BV in a cohort of women at high risk for STI/HIV in Uganda over 2 years of follow-up between 2008–2011.

**Methods:**

1027 sex workers or bar workers were enrolled and asked to attend 3-monthly follow-up visits. Factors associated with prevalent BV were analysed using multivariate random-effects logistic regression. The effect of treatment on subsequent episodes of BV was evaluated with survival analysis.

**Results:**

Prevalences of BV and HIV at enrolment were 56% (573/1027) and 37% (382/1027), respectively. Overall, 905 (88%) women tested positive for BV at least once in the study, over a median of four visits. Younger age, a higher number of previous sexual partners and current alcohol use were independently associated with prevalent BV. BV was associated with STIs, including HIV. Hormonal contraception and condom use were protective against BV. Among 853 treated BV cases, 72% tested positive again within 3 months. There was no difference in time to subsequent BV diagnosis between treated and untreated women.

**Conclusions:**

BV was highly prevalent and persistent in this cohort despite treatment. More effective treatment strategies are urgently needed.

## Introduction

Bacterial vaginosis (BV) is a common vaginal condition among women in sub-Saharan Africa, with prevalence ranging from 6% to 58%.^1^ BV is characterised by a reduction in lactic acid-producing lactobacilli and concurrent increase in anaerobic bacteria. BV has been described as a dysbiosis, or a microbial imbalance, and has been associated with preterm delivery,^2^ pelvic inflammatory disease (PID)^3^ and sexually transmitted infections (STIs).^1^ ^4–6^ Furthermore, BV increases viral replication and vaginal shedding of HIV-1^7^ and herpes simplex virus (HSV)-2,^8^ facilitating transmission of these viruses.

BV manifests clinically as a thin, whitish, homogeneous vaginal discharge and the presence of an amine odour.^9^ However, BV is often asymptomatic; in a recent study in South Africa, 90% of BV cases were asymptomatic.^10^ Current recommendations are that symptomatic BV is treated with oral metronidazole 400–500 mg twice a day for 5–7 days;^11^ ^12^ however, in resource-limited settings, BV is often treated according to WHO recommendations for managing vaginal discharge syndrome (VDS) with 2 g of metronidazole in a single dose.^13^

Once treated, BV often recurs; treatment trials report cure rates of 80–90%, but recurrence rates of 43% within 3 months.^14^ However, few studies have reported the recurrence of BV in sub-Saharan Africa where BV prevalence is high. In a study carried out among 35-year-old to 65-year-old women in South Africa, 32% of women treated for BV had recurrent BV within 12 months.^15^ More research is needed to investigate the occurrence and recurrence of BV in key populations for HIV infection.

The high prevalence of BV with its numerous adverse sequelae and high recurrence rates is of increasing international concern, especially in settings with high rates of STIs and HIV. Reducing prevalence of BV has the potential to reduce acquisition of related infections.

Our study aims to describe the epidemiology of BV in a cohort of women at high risk for STI and HIV infection in Kampala, Uganda. We describe the prevalence of BV over a 24-month period, behavioural and biological factors associated with BV, and the effect of treatment on subsequent diagnosis of BV.

## Methods

### Study design, setting and participants

The study methods have been described previously.^16^ In brief, the Good Health for Women Project is an observational cohort of self-identified sex workers and women working in bars and other entertainment venues in southern Kampala. The study offers free general and reproductive healthcare for participants and their children aged under 5 years. Participants were recruited between 2008 and 2009 and asked to attend follow-up every 3 months. Genital samples were systematically collected for the first 24 months of follow-up.

Participants were included in the study if they were aged at least 18 years or considered a mature minor, involved in high-risk sexual activity and willing to participate in HIV counselling and testing. Participants attended an enrolment interview and clinical examination. At each visit, data were collected on socio-demographic factors, alcohol and drug use, intravaginal practices, sexual behaviour, contraception, pregnancy and current symptoms. Blood samples were obtained at every visit; genital sampling and a routine genital examination were carried out at all visits except at months 15 and 21 due to financial constraints. Genital symptoms were recorded at every visit, and participants with symptoms consistent with PID, VDS or genital ulcer disease (GUD) were treated syndromically according to the Ugandan Ministry of Health Clinical Guidelines for STI management.^17^ Participants treated for VDS and PID received 2 g of metronidazole in a single dose or 400 mg of metronidazole twice daily for 14 days, respectively.^17^ In accordance with guidelines, women with asymptomatic laboratory-diagnosed BV were not treated. Women with laboratory-diagnosed asymptomatic STIs were treated. All participants with confirmed HIV infection were referred to an HIV care centre.

### Laboratory testing

A vaginal swab was used to prepare a Gram-stained slide that was examined for vaginal yeast and for BV using Nugent score.^18^ A Nugent score of 0–3 indicated normal microbiota, 4–6 indicated intermediate microbiota and 7–10 indicated BV. Another vaginal swab was inoculated in an InPouch culture for *Trichomonas vaginalis* and read daily for motile trichomonads for 5 days or until positive. A cervical swab was used to test for *Neisseria gonorrhoeae and* *Chlamydia* *trachomatis* using the Roche Amplicor *C. trachomatis/N. gonorrhoeae* PCR test. A single HIV Abbott Determine HIV-1/2 rapid test was performed onsite at the GHWP laboratory. Negative results were given to the participant immediately. Positive samples were sent to the MRC/UVRI serology laboratory in Entebbe for confirmation using Vironostika Uniform II (plus O) and Murex HIV EIA tests performed in parallel. Discordant or equivocal tests were retested by western blot to resolve the status. HIV-negative participants were routinely retested at subsequent visits. Syphilis serology was assessed using a quantitative Biotec rapid plasma reagin (RPR) test and the *Treponema pallidum* haemagglutination test (TPHA). Active syphilis was defined if both RPR and TPHA tests were positive, and an RPR titre of ≥1:8 was considered high-titre active syphilis. HSV-2 serology was performed using the HSV Type 2-IgG Kalon ELISA.

### Statistical methods

Analyses were performed using Stata/SE, V.13.1 (StataCorp, College Station, Texas, USA). BV was considered as a binary outcome: samples with Nugent score of 0–6 were considered BV negative and samples with Nugent scores 7–10 were considered BV positive. We assessed trends in BV prevalence over the follow-up using logistic regression with random effects to account for within-woman correlation. ORs for trend (OR_trend_) were obtained to estimate the change in BV prevalence from one 3-monthly follow-up visit to the next.

For the risk factor analysis, we examined factors associated with prevalent BV throughout the study period using a repeated-measures analysis (random-effects logistic regression). p-Values were obtained using likelihood ratio tests. Incident HIV and HSV-2 infections were defined as a positive test after a previously negative test.

A multivariate logistic regression model was constructed using a modified hierarchical framework^19^ based on the following levels: (1) socio-demographic, (2) sexual and behavioural factors and (3) biological factors. Level 1 factors associated with BV (p<0.20) in the unadjusted model were included in an adjusted regression model and retained as level 1 variables if they remained independently associated with BV (p<0.10). Behavioural variables associated with BV (p<0.20), plus the retained level 1 variables, were then included in the multivariate model and retained as level 2 variables if they remained independently associated with BV after adjustment for the covariates in the model (p<0.10). Associations with level 3 factors were assessed in the same way.

We also examined the effects of treatment on subsequent BV separately for previously untreated cases and cases treated with metronidazole at the last visit. For this analysis, we adjusted for all variables retained in the framework described above.

Lastly, to investigate the effect of treatment on time to next BV episode, we used Cox regression analysis and restricted the data to participants who were diagnosed with BV at the enrolment visit. We compared participants who were untreated, treated with 2 g of metronidazole and treated with 400 mg of metronidazole for 14 days. A multivariate Cox regression model adjusted for all variables retained in the framework described above, and any reported genital symptoms. A Kaplan–Meier curve was used to illustrate the time to next diagnosis by treatment.

## Results

### Characteristics of study population at enrolment

Of the 1214 women screened, 1027 (85%) were eligible, consented and enrolled in the cohort. The median age of the cohort was 26 years, and the majority (89.3%) were under 35 years. Over 90% had some level of education, although few (10.3%) had completed higher than primary level. The majority (63.9%) were separated or divorced and most had children. At enrolment, 69% of women reported five or more partners in the last month. Two-thirds of participants reported at least five occasions of transactional sex in the past month. Most women (60%) reported consistent use of condoms with clients in the past month, but 5% reported never using them. At enrolment, 56% of participants were assessed as problem drinkers by the CAGE questionnaire.

Most women (94%) reported vaginal cleansing in the past three months. Over half used water and soap to cleanse (57%) and 40% reported cleansing more than three times a day. Over half (56%) reported intravaginal insertion at least once in the past three months, mostly using herbs or other traditional substances but also aerated drinks (eg, Coca-Cola), gels and saliva. Genital symptoms were common at enrolment (68%) and included dysuria (11%), genital itching (36%) or burning (21%), dyspareunia (22%), lower abdominal pain (30%), abnormal discharge (40%) or ulcers/blisters (12%); 61%, 23% and 7% were treated at enrolment for VDS, PID and GUD, respectively. Prevalence of *N. gonorrhoeae* infection was 13%, *C. trachomatis* was 9% and *T. vaginalis* was 17%. Seroprevalences of HSV-2 (80%) and HIV (37%) were high.

### BV prevalence over time and untreated BV

Of the 1027 women enrolled in the study, 69% were retained in follow-up at 24 months with a median of four visits per woman (range 1–7), amounting to a total of 5801 total visits. Of these, 5568 (96%) had an available Nugent score result and were included in the analyses. The per-visit BV prevalence was 57%, and 88% of the entire cohort had at least one positive BV result. Of the women who attended all seven clinical visits, more than half (58%) of the participants had a positive BV result for at least four of the seven visits. Of the 3184 visits with BV diagnosis, 2033 (64%) had no signs or symptoms corresponding to a diagnosis of VDS or PID documented, and therefore, remained untreated in line with current guidelines. However, of these untreated BV cases, about half (46%) reported at least one genital symptom and were not strictly asymptomatic.

Prevalence of BV at enrolment was 56%, and there was a small increase over time (OR_trend_=1.10; 95% CI 1.05 to 1.14 per 3-month period) (table 1). However, there was a decrease in prevalence of BV with any genital symptoms and treated BV over time (OR_trend_=0.48; 95% CI 0.45 to 0.52, OR_trend_=0.77; 95% CI 0.74 to 0.82, respectively). We carried out a sensitivity analysis by restricting the data to the 584 participant samples available at 24 months, but the prevalences and trends remained unchanged (see online supplementary table S1).

**Table 1:** Prevalence trends in bacterial vaginosis (BV) diagnosis over seven visits among those with Nugent results at each visit in a cohort of 1027 women at high risk in Kampala, Uganda (2008–2011)

	Enrolment	3 months	6 months	9 months	12 months	18 months	24 months			
	n=1027	n=886	n=792	n=777	n=759	n=743	n=584	OR_trend_*	p Value†	Trend
Normal	354 (35%)	330 (37%)	308 (39%)	298 (36%)	301 (40%)	262 (35%)	202 (35%)	1.00 (0.97 to 1.04)	0.87	↔
Intermediate	100 (10%)	73 (8%)	60 (8%)	49 (6%)	29 (4%)	27 (4%)	14 (2%)	0.79 (0.74 to 0.84)	<0.001	↓
BV	573 (56%)	483 (55%)	424 (54%)	450 (58%)	429 (57%)	454 (61%)	368 (63%)	1.07 (1.04 to 1.11)	<0.001	↑
BV treated with metronidazole‡	400 (70%)	270 (56%)	177 (42%)	138 (31%)	103 (24%)	41 (9%)	22 (6%)	0.78 (0.75 to 0.82)	<0.001	↓
BV with any genital symptoms§	402 (70%)	309 (64%)	243 (57%)	244 (54%)	227 (53%)	259 (48%)	159 (43%)	0.48 (0.45 to 0.52)	<0.001	↓

*OR for trend in BV prevalence from one 3-monthly follow-up visit to the next.

†Likelihood ratio (LR) test p values were used to examine whether the OR_trend_ were likely to be due to chance.

‡This is a subset of the BV cases; treatment includes any participants with the diagnosis of BV and who were treated with 2 g of metronidazole in a single dose or 400 mg of metronidazole twice daily for 14 days at that visit.

§This is a subset of the BV cases; ‘any genital symptoms’ included dysuria, genital itching or burning, dyspareunia, lower abdominal pain, abnormal discharge or ulcers/blisters. Genital itching or burning, dysuria and ulcers/blisters are not treated with metronidazole in the syndromic management algorithm.

### Factors associated with prevalent BV

Associations of BV with socio-demographic factors are shown in table 2. In adjusted analyses, risk of BV was higher for women who were younger (adjusted p-trend=0.004) and had lower levels of education (adjusted p-trend=0.01). BV was independently associated with behavioural factors including increasing number of lifetime partners (adjusted p-trend<0.001), any alcohol use in the past three months (aOR=1.30; 95% CI 1.09 to 1.56) and decreased reported use of condoms with paying clients in the past month (adjusted p-trend<0.001).

**Table 2:** Factors associated with bacterial vaginosis (BV) at all visits in a repeated-measures analysis of 1027 women at high risk in Kampala, Uganda (2008–2011)

	Visits with BV diagnosed/ number of visits (%)	Unadjusted OR (95% CI)	Adjusted OR (95% CI)
Total	3180/5569 (57.1%)		
**Socio-demographic factors** (level 1)*			
Age (years; n=5569)		p_trend_=0.008	p_trend_=0.004
14–24	1249/2111 (59.2%)	1	1
25–34	1623/2836 (57.3%)	0.89 (0.70 to 1.13)	0.87 (0.69 to 1.11)
35+	309/622 (49.7%)	0.56 (0.38 to 0.82)	0.54 (0.37 to 0.79)
Highest education (n=5569)		p_trend_=0.03	p_trend_=0.01
Higher than primary level	305/585 (52.1%)	1	1
Primary school completed	1246/2223 (56.1%)	1.19 (0.80 to 1.75)	1.21 (0.82 to 1.80)
Primary school not completed	1352/2321 (58.3%)	1.31 (0.89 to 1.94)	1.37 (0.93 to 2.03)
Never attended school	278/440 (63.2%)	1.81 (1.06 to 3.10)	1.91 (1.12 to 3.26)
Regular partner (n=5566)†		p=0.29	p=0.32
No	702/1273 (55.2%)	1	1
Yes	2478/4293 (57.7%)	1.10 (0.92 to 1.33)	1.09 (0.91 to 1.32)
Number of live births (n=5224)†		p_trend_=0.06	p_trend_=0.52
None	140/202 (69.3%)	1	1
1–2	1570/2736 (57.4%)	0.62 (0.34 to 1.16)	0.66 (0.36 to 1.24)
3–4	959/1715 (55.9%)	0.56 (0.30 to 1.06)	0.67 (0.35 to 1.28)
≥5	309/571 (54.1%)	0.49 (0.24 to 0.99)	0.63 (0.30 to 1.33)
**Sexual and behavioural factors** (level 2)‡			
Age of first sex (years; n=5357)†		p=0.15	p=0.76
≤14	1128/1881 (60.0%)	1	1
15–16	1182/2067 (57.2%)	0.87 (0.66 to 1.13)	0.93 (0.72 to 1.22)
17–18	603/1140 (52.9%)	0.69 (0.50 to 0.94)	0.85 (0.62 to 1.18)
≥19	152/269 (56.5%)	0.87 (0.51 to 1.51)	1.04 (0.60 to 1.79)
Number of lifetime sexual partners (n=5569)		p_trend_<0.001	p_trend_<0.001
<20	347/690 (50.3%)	1	1
20–50	428/841 (50.9%)	1.05 (0.69 to 1.61)	1.05 (0.69 to 1.60)
50+	184/327 (56.3%)	1.37 (0.79 to 2.39)	1.39 (0.80 to 2.54)
Can't remember	2222/3711 (59.9%)	1.73 (1.22 to 2.44)	1.79 (1.26 to 2.37)
Involvement in sex work (N=5564)†		p=0.14	p=0.47
Only reports sex work for employment	1040/1719 (60.5%)	1	1
Reports sex work and other employment	1789/3183 (56.2%)	0.86 (0.71 to 1.05)	0.90 (0.75 to 1.09)
Reports no sex work	348/662 (56.2%)	0.78 (0.60 to 1.02)	1.01 (0.72 to 1.43)
Number of paying sex partners in the past month (N=5569)		p=0.09	p=0.79
0–4	1005/1869 (53.8%)	1	1
5–49	1648/2806 (58.7%)	1.30 (1.01 to 1.67)	1.09 (0.83 to 1.43)
50+ or can't remember	528/894 (59.1%)	1.31 (0.93 to 1.84)	1.01 (0.70 to 1.46)
Use of condoms with paying partners in the last month (N=5562)†		p=0.002	p_trend_<0.001
Never	221/350 (63.1%)	1	1
Sometimes	259/404 (64.1%)	0.99 (0.68 to 1.43)	0.99 (0.68 to 1.44)
Most of the time	764/1223 (62.5%)	0.90 (0.65 to 1.24)	0.86 (0.63 to 1.19)
Always	1374/2511 (54.7%)	0.70 (0.51 to 0.94)	0.68 (0.50 to 0.92)
Not applicable§	560/1074 (52.1%)	0.65 (0.48 to 0.90)	0.68 (0.49 to 0.93)
Alcohol use in the last three months (N=5567)†		p=0.002	p=0.004
No	882/1705 (51.7%)	1	1
Yes	2298/3862 (59.5%)	1.33 (1.11 to 1.59)	1.30 (1.09 to 1.56)
Illicit drug use in the last three months (N=5569)		p=0.07	p=0.35
No	2406/4277 (56.3%)	1	1
Yes	775/1292 (60.0%)	1.20 (0.99 to 1.45)	1.10 (0.81 to 1.33)
Intravaginal cleansing in the last three months (N=5558)†		p=0.29	p=0.28
No	183/334 (54.8%)	1	1
Yes, with water only	1312/2346 (55.9%)	0.96 (0.68 to 1.36)	0.92 (0.65 to 1.30)
Yes, with soap	1679/2878 (58.3%)	1.09 (1.03 to 2.02)	1.05 (0.75 to 2.58)
High-frequency cleansing in the last three months (N=5217)†,¶		p=0.60	p=0.37
No	2096/3636 (57.7%)	1	1
Yes	892/1581 (56.4%)	0.96 (0.81 to 1.13)	0.93 (0.78 to 1.10)
Insertion of a substance in the last three months (N=5568)†,**		p=0.11	p=0.02
No	2129/3660 (58.2%)	1	1
Yes	1052/1908 (55.1%)	0.88 (0.75 to 1.03)	0.82 (0.69 to 0.97)
**Biological factors** (level 3)††			
Hormonal factors (contraception/pregnancy; N=5368)†		p=0.01	p=0.02
No contraception	1419/2338 (60.7%)	1	1
Oral contraceptive pill	321/365 (56.8%)	0.83 (0.56 to 1.23)	0.81 (0.56 to 1.17)
Depot progesterone injection	675/1309 (51.8%)	0.63 (0.47 to 0.85)	0.66 (0.50 to 0.86)
Other‡‡	557/955 (58.3%)	0.94 (0.68 to 1.30)	1.02 (0.75 to 1.38)
Pregnant	209/401 (52.1%)	0.61 (0.39 to 0.95)	0.73 (0.48 to 1.10)
Vaginal yeast (N=5567)†		p<0.001	p<0.001
No	3012/5158 (58.4%)	1	1
Yes	168/409 (41.1%)	0.44 (0.34 to 0.58)	0.45 (0.34 to 0.59)
*Trichomonas vaginalis* (N=5565)†		p=0.003	p=0.05
No	2728/4924 (55.4%)	1	1
Yes	450/641 (70.2%)	1.44 (1.14 to 1.84)	1.27 (1.00 to 1.70)
*Neisseria gonorrhoeae* (N=5502)†		p=0.001	p=0.05
No	2799/5012 (55.9%)	1	1
Yes	350/490 (71.4%)	1.54 (1.19 to 1.99)	1.30 (1.00 to 1.70)
*Chlamydia trachomatis* (N=5543)†		p=0.66	p=0.86
No	3010/5283 (57.0%)	1	1
Yes	160/260 (61.5%)	1.08 (0.77 to 1.52)	1.03 (0.73 to 1.46)
Syphilis (N=5527)†		p<0.001	p=0.001
Negative	2404/4395 (54.7%)	1	1
Past syphilis (TPHA+/rpr−)	332/540 (61.5%)	1.40 (1.00 to 1.97)	1.17 (0.84 to 1.63)
Positive:low titre (TPHA+/rpr<8)	311/450 (69.1%)	2.02 (1.43 to 2.85)	1.47 (1.04 to 2.09)
Positive:high titre (TPHA+/rpr≥8)	111/142 (78.2%)	3.02 (1.71 to 5.34)	2.95 (1.62 to 5.39)
Current herpes simplex virus (HSV)-2 status (N=5555)†		p<0.001	p=0.001
Negative	410/923 (44.4%)	1	1
Prevalent HSV	2743/4593 (59.7%)	2.23 (1.68 to 2.95)	1.69 (1.27 to 2.24)
Incident HSV§§	23/39 (59.0%)	2.41 (1.07 to 5.40)	2.07 (0.88 to 4.84)
Current HIV status (N=5372)†		p<0.001	p<0.001
Negative	1704/3355 (50.8%)	1	1
Prevalent HIV	1317/1986 (66.3%)	2.35 (1.89 to 2.95)	1.94 (1.54 to 2.44)
Incident HIV§§	23/31 (74.2%)	3.88 (1.44 to 10.46)	2.69 (0.98 to 7.37)

*Adjusted for age and level of education.

†The difference from the number of visits (N=5569) is due to missing data.

‡BV at all visits adjusted for age, level of education, number of lifetime partners, frequency of condom use with clients, any use of alcohol and intravaginal insertion (n=1027, observations=5561).

§Includes those who did not have a paying partner in the past month.

¶High-frequency cleansing defined as cleansing >3 times/day, restricted to visits with reported cleansing in the past three months.

**Insertion of any of herbs, washing powder, soda, gel/lotions/Vaseline, saliva, other substances into vagina.

††BV adjusted for age, level of education, number of lifetime partners, frequency of condom use with clients, any use of alcohol, intravaginal insertion, HIV, HSV-2, vaginal yeast, *T. vaginalis*, *N. gonorrhoeae*, syphilis, hormonal contraception/pregnancy (N=1006, observations=4937).

‡‡Includes condoms, withdrawal methods, natural methods, traditional methods.

§§First diagnosed at this visit.

TPHA, *Treponema pallidum* haemagglutination test.

BV was independently associated with biological factors after adjusting for socio-demographic and behavioural factors. The strongest positive association was observed for high-titre syphilis (aOR=2.93; 95% CI 1.62 to 5.39). BV was also independently associated with prevalent HIV (aOR=1.94; 95% CI 1.54 to 2.44) and HSV-2 infections (aOR=1.69; 95% CI 1.27 to 2.24), low-titre syphilis (aOR=1.47; 95% CI 1.04 to 2.09) and *N. gonorrhoeae* infection (aOR=1.30; 95% CI 1.00 to 1.70). In contrast, participants with vaginal yeast had a strongly reduced risk of BV (aOR=0.45; 95% CI 0.34 to 0.59). There was some evidence that pregnancy and hormonal contraception were associated with a reduced risk of BV, especially use of depot medroxyprogesterone acetate (DMPA) injections (aOR=0.66; 95% CI 0.50 to 0.86 vs no contraception).

### Effect of BV treatment on subsequent BV

There was a strong association between BV and having been diagnosed with BV at the previous 3-month visit, irrespective of whether the participant had received treatment (table 3). Women who had received treatment for their BV at a previous 3-month visit were four times more likely to test positive for BV than those with no previous BV diagnosis (aOR=4.35; 95% CI 3.34 to 5.66). Among 853 treated BV cases, 72% tested positive again within 3 months.

**Table 3:** The association of bacterial vaginosis (BV) with treated or untreated BV at the visit 3 months prior at all visits in a repeated measures analysis of 1027 women at high risk in Kampala, Uganda (2008–2011)

	Number of BV cases during follow-up n/N (%)	Unadjusted odds ratio (95% CI)	Adjusted OR (95% CI)*
*Treatment for BV at visit 3 months prior (N=3046)†*		p<0.001	p<0.001
No BV at previous visit	331/1106 (29.9%)	1	1
Intermediate Nugent score at previous visit	137/255 (54.7%)	2.72 (2.02 to 3.67)	2.42 (1.78 to 3.29)
Treated BV	616/853 (72.2%)	5.19 (3.98 to 6.75)	4.35 (3.34 to 5.66)
Untreated BV	611/832 (73.4%)	5.52 (4.23 to 7.21)	4.87 (3.74 to 6.34)

*BV adjusted for age, level of education, number of lifetime partners, frequency of condom use with clients, any use of alcohol, intravaginal insertion, HIV, herpes simplex virus-2, vaginal yeast*, T. vaginalis, N. gonorrhoeae*, syphilis, hormonal contraception/pregnancy (N=946, observations=4233).

**†**Observations excludes the enrolment, 18-month and 24-month visit.

**Figure 1:**
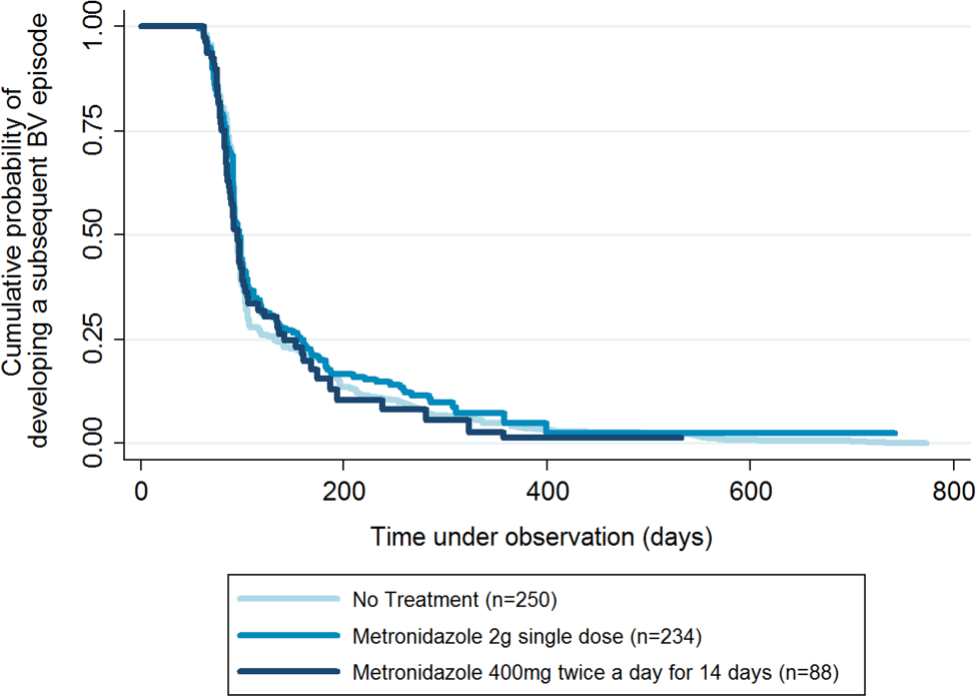
Kaplan–Meier survival curve demonstrating the time until subsequent diagnosis of bacterial vaginosis (BV) among 527 women diagnosed with BV at the enrolment visit in a cohort of 1027 women at high risk in Kampala, Uganda (2008–2011). This is over a 24-month period and stratified by treatment at the enrolment visit. The initial flat line reflects the intervals for scheduled clinic visits.

Among women diagnosed with BV at enrolment (N=527), there was no difference in time to subsequent BV diagnosis between no treatment versus treatment with 2 g of metronidazole in a single dose or 400 mg of metronidazole twice daily for 14 days (aHR: 0.87; 95% CI 0.70 to 1.07 and aHR: 1.02; 95% CI 0.75 to 1.39, respectively). Kaplan–Meier survival curves for next BV diagnosis are shown in figure 1. Of the women who had a subsequent BV episode, the overall median time to diagnosis was 99 days (range 57–796 days).

## Discussion

Our study showed a high prevalence of BV in a population at high risk of HIV and STIs—at any one visit, over half the participants tested were BV positive. In line with current management guidelines, half of all BV cases were not treated; yet there was no difference in time to a subsequent BV diagnosis between treated and untreated women with BV even after controlling for any genital symptoms. Given the known association of BV with STI/HIV, the high prevalence of BV and poor effectiveness of current treatment strategies pose a major public health problem in this population.

High prevalences of BV among female sex workers have been reported elsewhere in sub-Saharan Africa: 70% in South Africa and 40% in Kenya.^20^ ^21^ The strong association between BV and prevalent bacterial and viral STIs is consistent with previous studies.^1^ ^4–6^ While our analysis could not ascertain the direction of causation, the literature reports mechanisms for a causal relationship between BV and STI acquisition. Vaginal acidity has been shown to inhibit acquisition of *N. gonorrhoeae, C. trachomatis*, HSV-2 and HIV.^22–25^ During episodes of BV, lactobacilli, lactic acid and vaginal acidity are diminished, potentially increasing susceptibility to these infections. BV also induces an innate immune response in which cytokines associated with HIV acquisition are upregulated.^26^ In the classic transmission dynamics model of STIs, BV influences the transmission probability by increasing susceptibility to STIs. Importantly, half the participants had BV for over half their study visits, suggesting that a large proportion of participants may have long periods with increased susceptibility to STIs including HIV infection. In addition, BV also increases transmissibility of some viral STIs;^7^ ^8^ therefore, women with multiple episodes of BV may have prolonged periods of increased infectiousness.

A 7-day metronidazole oral regimen of 500 mg twice a day has been shown to provide an initial cure in 80–90% of BV cases (36); however, cure is seldom long term and recurrence is common.^15^ ^14^ In many resource-limited settings, women with symptomatic BV are managed according to national guidelines for the syndromic management of VDS and prescribed 2 g of metronidazole in a single dose. Previous research has shown that this is less efficacious than a 7-day course,^27^ and that recurrence is more common than with longer regimens.^28^ Our study suggested a risk of a subsequent diagnosis following treatment of >70%. While some participants were treated with a 14-day course of metronidazole as treatment for PID, the survival analysis showed no evidence of a difference in time to next BV diagnosis between no treatment, a single 2 g dose or 14-day course of metronidazole. For the latter treatment, non-adherence must be considered as a possible reason for persistent BV, especially in populations with high alcohol use.

Interestingly, while there was an increase in overall BV prevalence over 24 months, there was a reduction in BV treated with metronidazole and a more modest reduction of BV with any symptoms. This apparent paradox may be better understood within an ecological model of vaginal microbiota in which antibiotic perturbation causes shifts in the microbiota. Indeed, different BV-associated bacterial species and their metabolites have been shown to be associated with different clinical criteria;^29^ for example, *Atopobium vaginae* is associated with vaginal discharge and high pH, but not with an amine odour, while *Prevotella bivia* is associated with an amine odour and high pH, but not vaginal discharge. Therefore, treatment may clear bacteria responsible for some types of symptoms, while other types may remain and overgrow, causing a modified clinical presentation or asymptomatic recurrent infection. More research is needed to understand the dynamics of the vaginal microbiota in this population in relation to treatment and recurrence.

Our study suggests that hormonal contraception may be protective against BV, and this has also been shown elsewhere.^30^ Therefore, the promotion of hormonal contraception may also be a potentially effective intervention to prevent BV in this population. Lactobacilli metabolise glycogen from oestrogenised epithelial cells; therefore, exogenous oestrogen in combined contraceptives is thought to promote *Lactobacillus* colonisation. In progesterone-only contraceptives, such as DMPA, it has been theorised that the reduction in menstruation is protective against BV. However, caution is needed in view of recent meta-analysis showing increased risk of HIV associated with DMPA.^31^

Strengths of our study included its longitudinal design and the high retention rate in a high-risk population, providing a rich data source to examine multiple correlates of BV over time, although some bias due to losses to follow-up cannot be excluded. There were some further limitations to our study. Shifts in vaginal microbiota can be rapid, causing short episodes of BV and fast response to treatment.^32^ BV testing in this study was not designed to detect treatment success and was limited to 3-month and 6-month intervals; however, the rates of repeat positive tests were similar to 3-month recurrence rates observed in other treatment trials that included more intensive BV testing.^14^ Risk factors related to behaviours or infections of male partners were not measured in our study (eg, circumcision), and there may have been residual confounding if there were unmeasured or inaccurately measured risk behaviours.

In summary, for populations with high prevalence of BV and high HIV and STI incidence, more effective treatment strategies for BV are urgently needed. The development of innovative treatment strategies that effectively treat initial episodes of BV and prevent rapid recurrence should be a public health priority. Additionally, the potential role of hormonal contraceptives in the prevention of BV should be examined. Better treatments are required before carrying out trials to evaluate the impact of treatment of BV on the prevention of STIs, HIV or poor birth outcomes.

**Key messages**

- We found a very high prevalence (56%) of bacterial vaginosis (BV) among women who are at high risk for HIV and sexually transmitted infections (STIs).
- Of the women who were treated for BV, 72% had a second episode by 3 months.
- In line with current management guidelines, half of all BV cases were not treated; yet there was no difference in time to a subsequent BV diagnosis between treated and untreated women with BV.
- This is the highest rate of recurrent diagnosis reported to date and highlights the need to focus efforts on improved treatment of BV for the prevention of STIs and HIV in populations of high risk.

## Supplementary Material

Web supplement
