# Supplementary material for: Bacterial vaginosis among women at high risk for HIV in Uganda: high rate of recurrent diagnosis despite treatment
Source: Sex Transm Infect. 2015 Aug 7;92(2):142–8. doi: 10.1136/sextrans-2015-052160 (PMC4783330; doi:10.1136/sextrans-2015-052160)
Supplement: Web supplement [file sextrans-2015-052160-s1.pdf]

Table S1: Prevalence trends in BV diagnosis over 7 visits, restricted to 584 participants who had a Nugent score result at 24 months in a cohort of 1027 women at high risk in Kampala, Uganda (2008-2011)

	**Enrolment n=584**	**3 mths n=552**	**6 mths n=516**	**9 mths n=521**	**12 mths n=520**	**18 mths n=534**	**24 mths n=584**	**OR_{trend}[a]**	**p-value[b]**	**Trend**
Normal	203 (35%)	205 (37%)	201 (39%)	184 (35%)	207 (40%)	183 (34%)	202 (35%)	0.99 (0.95, 1.03)	0.60	↔
Intermediate	60 (10%)	49 (9%)	39 (8%)	37 (7%)	14 (3%)	19 (4%)	14 (2%)	0.77 (0.71, 0.82)	<0.001	↓
BV	321 (55%)	298 (54%)	276 (54%)	300 (58%)	299 (58%)	332 (62%)	368 (63%)	1.10 (1.05, 1.14)	<0.001	↑
BV treated with metronidazole[c]	239 (74%)	177 (59%)	135 (49%)	103 (34%)	88 (29%)	34 (10 %)	22 (6%)	0.77 (0.74, 0.82)	<0.001	↓
BV with any genital symptoms[d]	235 (73%)	196 (66%)	161 (58%)	167 (56%)	159 (53%)	165 (50%)	159 (43%)	0.48 (0.45, 0.52)	<0.001	↓

Legend:

a=OR for trend in BV prevalence from one three monthly follow-up visit to the next

b= Likelihood ratio (LR) test p-values were used to examine whether the OR_{trend} were likely to be due to chance

c=This is a subset of the BV cases; treatment includes any participants with the diagnosis of BV and who were treated with 2 grams of metronidazole in a single dose or 400 mg of metronidazole twice daily for 14 days at that visit

d=This is a subset of the BV cases; “Any genital symptoms” included dysuria, genital itching or burning, dyspareunia, lower abdominal pain, abnormal discharge, or ulcers/blisters. Genital itching or burning, dysuria and ulcers/blisters are not treated with metronidazole in the syndromic management algorithm.
